# Disparities in diabetic foot examinations: a cross-sectional analysis of the behavioural risk factor surveillance system

**DOI:** 10.1017/S1463423624000392

**Published:** 2025-03-26

**Authors:** Kristyn Robling, Kristen McPherson, Douglas Nolan, Benjamin Greiner, Micah Hartwell

**Affiliations:** 1 Office of Medical Student Research, Oklahoma State University College of Osteopathic Medicine at the Cherokee Nation, Tahlequah, OK, USA; 2 Tribal Health Affairs, Oklahoma State University College of Osteopathic Medicine at the Cherokee Nation, Tahlequah, OK, USA; 3 Department of Family Medicine, Oklahoma State University College of Osteopathic Medicine at the Cherokee Nation, Tahlequah, OK, USA; 4 Department of Internal Medicine, University of Texas Medical Branch, Galveston, TX, USA; 5 Department of Psychiatry and Behavioral Sciences, Oklahoma State University Center for Health Sciences, Tulsa, OK, USA

**Keywords:** BRFSS, diabetes mellitus, diabetic foot examination, foot ulcers, preventive care

## Abstract

**Aim:**

This study aimed to identify how frequent poor mental health days, a depressive disorder diagnosis, frequent poor physical health days, or physical inactivity affect annual foot examinations in individuals with diabetes.

**Background:**

Diabetes mellitus (DM), particularly type 2, is a growing problem in the United States and causes serious health complications such as cardiovascular disease, end-stage renal disease, peripheral neuropathy, foot ulcers, and amputations. There are guidelines in place for the prevention of foot ulcers in individuals with diabetes that are not often followed. Poor mental health and poor physical health often arise from DM and contribute to the development of other complications.

**Methods:**

We performed a cross-sectional analysis of the 2021 Behavioural Risk Factor Surveillance System dataset to determine the relationship between annual foot examinations and frequent poor mental health days, a depressive disorder diagnosis, frequent poor physical health days, or physical inactivity using a bivariate logistic regression model. The regression model was controlled for age, sex, race/ethnicity, health insurance, level of education, current smoking status, and Body Mass Index (BMI) category.

**Findings:**

Our results showed that 72.06% of individuals with frequent poor mental health days received a foot check, compared with 76.38% of those without poor mental health days – a statistically significant association (AOR: 1.25; 95% CI: 1.09–1.43). Of those reporting a sedentary lifestyle, 73.15% received a foot check, compared with 77.07% of those who were physically active, which was also statistically significant (AOR: 1.31; 95% CI: 1.14–1.49). Although individuals reporting depressive disorder diagnoses and frequent poor physical health days had lower rates of foot examinations, these results were not statistically significant. To reduce rates of foot ulcers and possible amputations, we recommend the implementation of counselling or support groups, increased mental health screening, educational materials, or exercise classes.

## Introduction

Diabetes mellitus (DM) is on the rise in the United States, particularly type 2 DM, posing serious health risks for over 31 million people as of 2019 (Saeedi *et al.*, 2019). These health risks can be divided into macrovascular and microvascular complications with the former including cardiovascular disease, stroke, and peripheral vascular disease – the latter often presents as end-stage renal disease, retinopathy, and peripheral neuropathy (Harding *et al.*, 2019). Peripheral neuropathy may be present in up to 50% of those with diabetes and can lead to its own complications, including severe pain, inability to ambulate, and development of foot ulcers.

Foot ulcers can lead to a higher risk of falls and fractures, frequent infections, hospitalizations, increases in depression, and amputation of the foot (Dixit and Maiya, 2014; Adeyemi *et al.*, 2021). In the United States, over 60% of non-traumatic amputations were lower extremity amputations in 2003 (Bonner, Foster, and Spears-Lanoix, 2016), and 20% of hospital admissions were due to an infected diabetic foot ulcer in 2007 (Edmonds, Manu, and Vas, 2021). Owing to these serious complications, there are guidelines in place for people with diabetes and physicians to follow for better prevention of diabetic foot ulcer development. The Center for Disease Control (CDC) has established guidelines for physicians to follow, including risk assessment, patient self-care, diabetes management and education, and annual diabetic foot examinations (*How to promote foot health for people with diabetes*, 2022). However, these guidelines are somewhat limited in breadth, so there are others available that offer further specificity.

The International Working Group on the Diabetic Foot (IWGDF), an organization that creates detailed sets of recommendations, provides deeper insight into diagnosis, prognosis, and management. They identify five key elements of prevention: education, appropriate footwear, risk factor treatment, identification of the at-risk foot, and regular inspection. Education is particularly important in assisting the patient with self-prevention and should cover how to self-inspect the foot, identify temperature changes, notify a healthcare provider of damaged skin, choose proper footwear, and utilize proper hygiene practices. Appropriate footwear is key to preventing foot trauma that can lead to ulceration and should include properly fitting shoes that have a wide toe box to prevent compression and ensure proper loading of the foot. Treating risk factors for ulceration can include removing any callus, protecting blisters, treating fungal infections, or providing appropriate toenail care. Identifying the at-risk foot involves checking for a history of previous ulcers, pedal pulses, loss of sensation, pressure perception, and vibration perception. Once the foot has been initially assessed, they recommend performing regular inspections at least once a year to identify new changes in the status of the foot (Schaper *et al.*, 2020). It is estimated that by following these detailed guidelines thoroughly, up to 75% of foot ulcers can be prevented. Despite this predicted high success rate, thorough adherence to the IWGDF guidelines appears to be poorly implemented. A few barriers to care have been suggested to explain why (Van Netten, Woodburn, and Bus, 2020).

Research indicates that there is a trend towards focusing on ulcer healing over prevention in the medical field. One possible explanation is the lack of financial gain from preventative ulcer care. Additionally, there is a greater availability of specialized health care to guide ulcer healing compared with preventative modalities. Patients are transitioned to community-based health care following ulcer resolution, where physicians’ ability to educate patients on self-care is more limited by time and resource availability (Van Netten, Woodburn, and Bus, 2020). Furthermore, there are several non-systemic barriers that people experience when it comes to self-prevention. Many people with diabetes have low health literacy, which limits their understanding of medical treatments and behavioural modifications to better control their diabetes and may often contribute to worsening symptoms (Gazmararian, Ziemer, and Barnes, 2009). These individuals may often find it difficult to routinely check their feet (Pérez-Panero *et al.*, 2019) – nearly two-thirds of people with diabetes reported rarely or never examining their feet (Coffey, Mahon, and Gallagher, 2019). Appropriate footwear recommendations are also frequently neglected due to low perception of ulceration risk, appearance of therapeutic footwear, or fear of drawing attention to their medical diagnosis (Paton *et al.*, 2014). Moreover, some people with diabetes avoid medical visits, citing low perception of symptom severity, medical mistrust, or previous negative experiences. Some avoid care for fear of worsening prognosis (Taber, Leyva, and Persoskie, 2015). Emotional factors such as depressive disorders play a role in self-care as well, with nearly a third of patients with diabetes experiencing depressive symptoms. This contributes to an increase in morbidity and mortality as well as decreased quality of life. It is suggested that hardships due to diagnosis, such as personal expenses or declining health, are major factors that may be responsible for poor mental health (Lustman and Clouse, 2005). This decline in mental health is associated with a two-fold increase in foot ulcer incidence (Williams *et al.*, 2010).

Given that DM is on the rise and that foot ulcers may lead to serious health complications that are otherwise preventable by following guidelines, we sought to identify potential factors that are associated with poor routine foot inspection among those with diabetes. As mentioned, there is evidence that poor mental health and many physical complications can result from diabetes. Therefore, we assessed data from the 2021 Behavioural Risk Factor Surveillance System (BRFSS) to investigate the relationship between routine foot checks and frequency of poor mental and physical health days, as well as diagnosis of a depressive disorder and reported exercise. We hypothesized that poor mental health, poor physical health, diagnosis of depressive disorder, and lack of exercise would be associated with a decrease in examination of feet.

## Subjects, materials, and methods

### Eligibility/inclusion

For our study, we conducted a cross-sectional analysis of the 2021 BRFSS to assess the relationship between routine foot checks and frequency of poor mental health days, diagnosis of a depressive disorder, poor physical health days, and reported exercise. BRFSS is a system of telephone surveys used to identify state-level data from all 50 states on health risk behaviours, chronic medical conditions, use of preventative services, and demographics (*BRFSS*, 2022). BRFSS surveys approximately 500,000 individuals in the United States annually. BRFSS data is publicly available and does not include individually identifying information; therefore, it does not meet the requirements for human subjects research. As such, this investigation was not submitted for ethics review.

### Sample inclusion

We included respondents as having a diagnosis of diabetes if they answered ‘yes’ to the prompt ‘Has a doctor, nurse, or other health professional ever told you that you had any of the following – diabetes?’ Those who responded with ‘Yes, but female told only during pregnancy’, ‘no’, ‘no, pre-diabetes or borderline diabetes’, ‘don’t know/not sure’, ‘refused’, or whose response was marked as missing were excluded from this study.

Further, to assess foot care, we excluded individuals who did not respond or refused to provide an answer to the question: About how many times in the past 12 months has a health professional checked your feet for any sores or irritations? Responses included in this study were from 0 times in the past 12 months to 76 times. This was coded as a binary variable to report adherence to CDC guidelines of having a health professional check feet at least once per year.

### Variables of interest

Variables assessed in relation to the frequency of foot checks were the number of poor mental health days, number of poor physical health days, diagnosis of depressive disorder, and physical activity. To assess the number of poor mental health days, we used the prompt ‘Now thinking about your mental health, which includes stress, depression, and problems with emotions, for how many days during the past 30 days was your mental health not good?’ BRFSS provides categorical variables as having 0–13 days or 14 or more (frequent poor mental health days) in line with previous research indicating ≥14 days as a significant indicator of poor mental health (Dwyer-Lindgren *et al.*, 2017). The number of poor physical health days was extracted from the prompt ‘Now thinking about your physical health, which includes physical illness and injury, for how many days during the past 30 days was your physical health not good?’ BRFSS provides categorical variables as having 0–13 days or 14 or more (frequent poor physical health days) in line with previous research indicating ≥14 days as a significant indicator of poor physical health (Dwyer-Lindgren *et al.*, 2017). Diagnosis of depressive disorder was obtained in response to the question ‘Has a doctor, nurse, or other health professional ever told you that you had any of the following – a depressive disorder (including depression, major depression, dysthymia, or minor depression)?’ Physical activity was assessed with the prompt ‘Adults who reported doing physical activity or exercise during the past 30 days other than their regular job’.

### Demographic variables

Sociodemographic variables from BRFSS that were related to our investigation were extracted to use as controls in our study. These variables included (1) age, (2) sex (male or female), (3) race/ethnicity (non-Hispanic White, non-Hispanic Black, non-Hispanic Asian, non-Hispanic American Indian/Alaskan Native, Hispanic, or non-Hispanic other race), (4) health insurance (insured or uninsured), (5) level of education (did not graduate high school, high school, attended college/technical school, graduated college/technical school), (6) current smoking status (yes or no), and (7) Body Mass Index (BMI) category (underweight, normal weight, overweight, obesity). Responses of ‘don’t know’, ‘refused’, or those marked as missing were not included in our analysis.

### Statistical analysis

First, we reported sample size (n), population estimate (N), overall, and between individuals who did and did not have any foot checks by healthcare professionals (as a binary variable). We additionally report this breakdown by demographic variables. Next, we report the percentages of individuals with and without having foot checks by those having frequent (≥14) poor mental health days and whether they reported having a diagnosis of depression and also among those having frequent (≥14) poor physical health days or having performed physical activity in the past 30 days. We then used a bivariate logistic regression model to determine associations, via odds ratios, between having had foot checks and these mental and physical health variables. We then provided adjusted estimation models controlling for age, sex, race/ethnicity, health insurance, level of education, current smoking status, and BMI category. We used Stata 16.1 (StataCorp LLC, College Station, TX) to analyse the selected BRFSS data. Alpha was set at 0.05 for all analyses.

## Results

### Study sample

There were 438,693 respondents who completed the BRFSS survey. After excluding those without diabetes and those with diabetes who did not respond or refused to provide an answer to whether or not their feet had been checked, our sample (n) consisted of 21,372 people. Applying sampling weights resulted in an estimated population (N) of 12,757,834. Of this sample, 16,121 (75.54%, N = 9,637,131) reported having their feet checked by a medical professional at least once within the past year, while 5,251 (24.46%, N = 3,120,703) reported no foot checks. Demographics of the sample are provided in Table 1.

**Table 1.** Demographics of individuals with diabetes by whether they had had their feet checked by a healthcare professional in the past 12 months (n = 21372; N = 12757834)

	Foot check by HCP			
	Total n (%)	Yes n (%)	No n (%)	Test, *P*
Overall	21372 (100)	16121 (75.54)	5251 (24.46)	
Age group				
18–24	83 (0.51)	43 (42.81)	40 (57.19)	*X* ^2^ = 14.97, < .0001
25–34	373 (2.12)	232 (59.5)	141 (40.5)	
35–44	1059 (6.15)	705 (66.07)	354 (33.93)	
45–54	2670 (15.10)	1935 (72.96)	735 (27.04)	
55–64	5121 (25.12)	3855 (75.49)	1266 (24.51)	
65+	12066 (51.00)	9351 (78.46)	2715 (21.54)	
Sex				
Male	10543 (50.29)	8126 (76.82)	2417 (23.18)	*X* ^2^ = 6.22,. 013
Female	10829 (49.71)	7995 (74.24)	2834 (25.76)	
Race/ethnicity				
White	15461 (68.28)	11794 (76.34)	3667 (23.66)	*X* ^2^ = 7.91, < .0001
Black	2511 (14.92)	1967 (79.51)	544 (20.49)	
Asian	260 (1.52)	169 (70.04)	91 (29.96)	
AI/AN	694 (1.45)	497 (73.1)	197 (26.9)	
Hispanic	1737 (11.17)	1196 (67.56)	541 (32.44)	
Other	709 (2.67)	498 (70.55)	211 (29.45)	
Has insurance?				
Yes	20016 (95.32)	15257 (76.79)	4759 (23.21)	*X* ^2^ = 51.24, < .0001
No	641 (4.68)	350 (53.75)	291 (46.25)	
Education level				
< HS	1835 (9.91)	1266 (69.29)	569 (30.71)	*X* ^2^ = 12.24, < .001
HS	6340 (29.01)	4770 (74.83)	1570 (25.17)	
Some college	6545 (29.86)	4997 (76.85)	1548 (23.15)	
College Graduate	6573 (31.23)	5033 (76.87)	1540 (23.13)	
Currently smoking				
No	17934 (87.39)	13666 (76.2)	4268 (23.8)	*X* ^2^ = 12.24, < .001
Yes	2530 (12.61)	1776 (70.82)	754 (29.18)	
Overweight or obesity				
No	2711 (13.76)	1990 (71.08)	721 (28.92)	*X* ^2^ = 8.90, .003
Yes	16762 (86.24)	12749 (76.33)	4013 (23.67)	
Years since diabetes diagnosis				
M (SD)	14.01 (11.50)	15.15 (11.53)	11.68 (10.85)	*t* = −11.11, <.0001

*Note:* HCP = Health Care Professional.

Of individuals between the ages of 18 to 24, 42.81% had their feet checked; however, with increasing age, the proportion of respondents who had their feet checked increased with 78.46% of those aged 65+ completing annual foot examinations (*X* ^2^ = 14.97, < .0001). Males and females had similar responses, with 76.82% of males having completed a foot check with a healthcare professional compared to 74.24% of females (*X* ^*2*^ = 6.22, .013). The range of individuals having completed a foot check was lowest among individuals identifying as Hispanic (67.56%) and highest among those who are Black (79.51%; *X* ^*2*^ = 7.91, < .0001). People with insurance reported a higher prevalence of foot checks (76.79%) compared to those who did not have insurance (53.75%; *X* ^*2*^ = 51.24, < .0001). Rates of foot checks were similar among those with a high school degree or who attended/graduated college; however, there was a 5.54% difference between those who did not graduate high school and those who did and a 7.58% difference between those who did not graduate high school and those who graduated college (*X* ^*2*^ = 12.24, <.001). Of those who are current smokers, there was a decrease in foot checks by 5.38% compared to those who do not smoke – 70.82% of smokers reported having foot checks compared to 76.2% of non-smokers (*X* ^*2*^ = 12.24, <.001). Individuals with a BMI greater than 25 had a higher prevalence of annual foot checks (76.33%) compared to those with a BMI less than 25 (71.08%; *X* ^*2*^ = 8.90, .003). Individuals who had a foot check in the past year, on average, were diagnosed with diabetes 15.15 (SD = 11.53) years ago, while those not having their feet checked had a shorter span of time since their diagnosis (M = 11.68; SD = 10.85; t = -11.11, <.0001).

### Depressive disorders and poor mental health days

Among our sample, 25.15% of respondents reported having been diagnosed with a depressive disorder. Among those with a depressive disorder, 72.93% reported annual foot checks compared with 76.42% of those with no depressive disorder; however, the observed association was not statistically significant (AOR: 1.13; 95% CI: 0.98–1.30). Within our sample, 15.75% of individuals reported having frequent (14+) poor mental health days. Among these individuals, 72.06% reported having a foot check compared to 76.38% who were classified as not having frequent poor mental health days. Thus, those who reported frequent poor mental health days were less likely to have their feet checked annually than those without frequent poor mental health days – a statistically significant observation (AOR: 1.25; 95% CI: 1.09–1.43). These values can be found in Table 2.

**Table 2.** Prevalence of foot checks among individuals with diabetes by mental and physical health variables, and the likelihood of *not* having feet checked

	Foot Check in the past 12 months			Logistic regression	
	Total n (%)	Yes n (%)	No n (%)	OR (95% CI)	AOR (95% CI)
Depressive disorder diagnosis					
No	16093 (74.85)	12239 (76.42)	3854 (23.58)	1 (Ref)	1 (Ref)
Yes	5177 (25.15)	3803 (72.93)	1374 (27.07)	1.2 (1.07–1.35)	1.13 (0.98–1.30)
Frequent poor mental health days (>14 days of the past 30 days)					
No	17822 (84.25)	13599 (76.38)	4223 (23.62)	1 (Ref)	1 (Ref)
Yes	3147 (15.75)	2238 (72.06)	909 (27.94)	1.25 (1.09–1.44)	**1.25 (1.09–1.43)**
Sedentary (no physical activity in the past 30 days)					
No	12938 (61.29)	9952 (77.07)	2986 (22.93)	1 (Ref)	1 (Ref)
Yes	8370 (38.71)	6122 (73.15)	2248 (26.85)	1.23 (1.10–1.38)	**1.31 (1.14–1.49)**
Frequent poor physical health days (>14 days of the past 30 days)					
No	15691 (75.08)	11899 (76.04)	3792 (23.96)	1 (Ref)	1 (Ref)
Yes	5042 (24.92)	3767 (74.85)	1275 (25.15)	1.07 (0.94–1.21)	1.08 (0.94–1.25)

Adjusted model controls for age, sex, time since diabetes diagnosis, race/ethnicity, insurance coverage, education, smoking status, and overweight/obesity. The significance for the values that are in bold is *P* < 0.01.

### Physical inactivity and poor physical health days

Among our sample, 38.71% of individuals reported physical inactivity. Of individuals who reported sedentary lifestyles, 73.15% had annual foot examinations compared to 77.07% of those who reported being active. Thus, those who reported physical inactivity were less likely to have their feet checked annually than those who reported physical activity. This observed association was statistically significant (AOR: 1.31; 95% CI: 1.14–1.49). Within our sample, 24.92% of individuals reported having frequent (14+) poor physical health days. Among these individuals, 74.85% reported having an annual foot check compared to 76.04% of those without frequent poor physical health days; however, the observed association was not statistically significant (AOR: 1.08; 95% CI: 0.94–1.25). These values can be found in Table 2.

## Discussion

While there are many studies that investigate how those with diabetes utilize health care, there is currently little known about how those with diabetes utilize preventive foot examinations. This manuscript contributes to the limited knowledge around foot examinations among those with diabetes in the hopes that it will guide physicians and patients in their healthcare approach. Our investigation found that among individuals with diabetes, those with frequent poor mental health and a sedentary lifestyle were significantly less likely to report having their feet checked by a healthcare professional in the past 12 months. Further, rates of foot checks were found to be lower among individuals who were female, Hispanic, lacked insurance, did not complete high school, were currently smoking cigarettes, had a BMI < 25, or were less than 35 years of age. Being diagnosed with a depressive disorder or having poor frequent physical health days resulted in fewer annual examinations; however, these results were not found to be statistically significant. Our results suggest that motivation to care for feet may be decreased in those who have poor mental health or who are sedentary, whether that be due to physical inability or lack of desire to exercise.

### Foot care, mental health, and physical activity

An annual foot examination by a healthcare professional is recommended for those with diabetes by multiple organizations (Schaper *et al.*, 2020; *How to promote foot health for people with diabetes*, 2022). However, our results indicate that those with frequent poor mental health days are less likely to get these annual examinations. Individuals with mental health complications may have an increase in perceived or actual barriers to care and forgo seeking medical evaluation (Coombs *et al.*, 2021), potentially including foot checks. Additionally, poor mental health is often associated with poor health literacy (Lincoln *et al.*, 2006), which could result in a decreased understanding of the importance of annual foot checks. Individuals with comorbid diabetes and mental health disorders may be less likely to follow treatment regimens or guidelines – leading to higher susceptibility of physical complications (Ducat *et al.*, 2015). These complications, such as foot ulcers, can lead to worsening mental and physical health symptoms – decreasing overall health (Hoban *et al.*, 2015). While our research showed a significant association between rates of foot checks and frequent poor mental health days, the same was not found for those with a depressive disorder diagnosis. One possible explanation for this is a lack of diagnoses among those with poor mental health. Depressive disorders and other mental health disorders often go undiagnosed and untreated in individuals with diabetes (Balhara, 2011); however, treatment decreases health risks and negative outcomes (Ducat, Philipson, and Anderson, 2014). Thus, individuals with a depressive disorder diagnosis likely receive treatment and experience better health outcomes, including receiving annual foot examinations. These results support the need for increased mental health screening among individuals with diabetes, the option for counselling or support groups, or integrated mental/behavioural health services in diabetes care.

The juxtaposition of significance between the associations of foot checks and being sedentary (significant) and frequent poor physical health days (non-significant) may indicate that there is lower motivation to sustain or improve health through self-care, rather than physical limitations. This is supported by a 2017 review that reported that lower rates of exercise among individuals with diabetes may result from a lack of motivation, unfamiliarity with appealing forms of physical activity, comorbidities, psychological barriers, or inadequate education on exercise recommendations (Jenkins and Jenks, 2017). Given exercise reduces health complications in those with diabetes, the American Diabetes Association (ADA) recommends partaking in aerobic exercise (150 minutes per week) and resistance training at least every other day (Colberg *et al.*, 2016); however, adults with diabetes report low levels of physical activity and are frequently sedentary for at least 70% of the day (Kennerly and Kirk, 2018). The decreased likelihood of undergoing an annual foot examination for those who reported a sedentary lifestyle may be explained by the utilization of healthcare services. Individuals who report physical activity are more likely to utilize annual physical check-ups (Kobayashi *et al.*, 2021). Additionally, individuals who are inactive use inpatient services 38% more (Sari, 2009) – potentially due to health complications that could be avoided by using preventative services more frequently.

### Demographics and foot care

Although similar in responses, we found that men were more likely to obtain annual foot examinations than women. Conversely, other research has found that men are often less likely to utilize healthcare visits and do not partake in preventive care as frequently as women do (Pinkhasov *et al.*, 2010). Individuals who identified as Hispanic were found to have the lowest rates of annual foot examinations, while those who were Black were found to have the highest rates. Health care for Hispanic and Black individuals has historically been of lower quality than that for White individuals (Bulatao, Anderson and National Research Council (US) Panel on Race, Ethnicity, and Health, 2004); however, Black individuals with diabetes are 1.98 times more likely to require a lower leg amputation due to foot ulcer complications (Miller *et al.*, 2022) and thus are more likely to utilize preventive care (Littman *et al.*, 2020).

Our results showed that those with a BMI < 25, who were currently smoking cigarettes, or who were younger than 35 were less likely to undergo a foot check. Individuals with a high BMI utilize healthcare services and preventive care more frequently than those with a BMI < 25 due to a higher risk for health complications (Bertakis and Azari, 2006). Individuals who were currently smoking cigarettes were less likely to have their feet checked, which may be due to an overall decreased utilization of preventive services among this group (Vander Weg, Howren, and Cai, 2012). Young adults are the least likely age group to utilize medical care and preventive care and frequently do not receive preventive counselling when visiting the doctor (Ozer *et al.*, 2012). Low rates of preventive services may be due to no insurance (Cantor *et al.*, 2012) or that perceptions of health are more positive with younger age (Giacomozzi *et al.*, 2020).

### Implications and recommendations

Certain demographic factors, sedentary lifestyles, and frequent poor mental health days may contribute to a decrease in diabetes foot care. Proper foot care and annual examinations are imperative for preventing the development of foot ulcers, gangrene, or amputations (Cousart and Handley, 2017). Sufficient education is an additional modality for patients to support their own care and prevent complications themselves (Al-Wahbi, 2010). To prevent foot ulcers moving forward, we recommend physicians continue to follow current guidelines and ensure every patient with diabetes gets an annual foot examination. In an effort to improve physician compliance as well, we suggest the implementation of a quality metric design for foot examinations. Physicians are more likely to perform a thorough diabetic work-up or provide education to their patients with a financial incentive in place (Beaulieu and Horrigan, 2005). It is vital that patients are properly educated about health complications and guidelines for self-prevention using either CDC materials or other thorough educational items. Educational sessions over a set time period that utilize PowerPoint presentations and handouts with material from the ADA are an effective way to increase patient knowledge of diabetes foot care (Gor and Fnp-C, 2021). A clinical trial is currently investigating what method of delivery is most effective for diabetes foot care, including watching a video, reading a leaflet in-office, or taking a leaflet home (*Educational Interventions on Diabetic Foot Care*, no date). This study will help guide physicians towards the best modality for educating patients. It would also be beneficial to implement mental health services for those with diabetes, increase mental health screenings at each healthcare visit, and offer counselling services or support groups to help individuals with diabetes improve their mental health. We also recommend thorough education on exercise and how it may benefit those with diabetes, as well as the implementation of exercise groups or programmes specifically designed for diabetes care. The information in this manuscript can be used to guide individual practice, but it can also be used to influence current policy. Such changes may include creating policies based on the recommendations we have listed for providers to follow. Policy changes may serve to further incentivize providers to provide foot examinations or investigate why their patients are not receiving them. By utilizing a multidisciplinary approach that involves physicians, podiatrists, psychiatrists/psychologists, physical therapists, pharmacists, nurses, and diabetes specialists, a plan can be designed to best optimize each individual’s health.

### Strengths and limitations

This study had several strengths and limitations. First, we utilized a cross-sectional design, preventing the ability to infer causal relationships. Second, all variables were self-reported, introducing the possibility of inaccuracies or response bias. Third, BRFSS did not ask if respondents developed an ulcer, preventing identification of the effectiveness of the foot checks. Fourth, BRFSS utilizes landline and telephone data collection, preventing collection from populations without access to these devices. Fifth, this research utilizes only data from the United States; although this limits the breadth of this research, we believe this information is vital to international providers and patients as well due to the global prevalence of diabetes being predicted to reach over 10% by 2030 (Saeedi *et al.*, 2019). Conversely, a strength of this study is the large sample size provided by BRFSS with a diversified profile that effectively represents a national population. Future research should identify the effectiveness of ulcer prevention in those who reported annual foot examinations.

## Conclusions

Little is currently known about how those with diabetes utilize foot ulcer examinations. This research was conducted to improve knowledge surrounding this topic and help guide physicians and patients in health care. This study found that a sedentary lifestyle and frequent poor mental health days in individuals with diabetes were associated with a decrease in annual foot examinations by a healthcare provider. Given the serious health complications that can arise from improper foot care, such as ulcers or amputations, we recommend strict adherence to current preventive guidelines and an increase in patient education. We also recommend the implementation of improved mental health resources for individuals with diabetes as well as a variety of resources to increase physical activity. These adjustments will encourage improved foot care for those with diabetes and reduce rates of foot-derived complications.
